# Donor Site Outcomes Following Autologous Breast Reconstruction with DIEP Flap: A Retrospective and Prospective Study in a Single Institution

**DOI:** 10.1177/22925503241255118

**Published:** 2024-05-20

**Authors:** Stacy Fan, Stephanie Kim, Kaveh Farrokhi, Dianna Deng, Laryssa Laurignano, Devin Box, Aaron Grant, Sarah Appleton, Tanya DeLyzer

**Affiliations:** 1 Division of Plastic and Reconstructive Surgery, 6221 Western University, London, Ontario, Canada; 2 Schulich School of Medicine & Dentistry, 70384 Western University, London, ON, Canada

**Keywords:** Autologous breast reconstruction, donor site outcomes, DIEP breast reconstruction, reconstruction mammaire autologue, résultats au foyer du donneur, reconstruction mammaire par l’AÉIP

## Abstract

**Background:** The deep inferior epigastric perforator artery (DIEP) free flap is the most commonly performed autologous breast reconstruction. Despite the relative reduction in donor site complications compared to nonmuscle-sparing options, there is still high morbidity associated with this surgery. The purpose of this study is to compare outcomes and complications at our institution and to discuss potential quality improvement initiatives. **Methods:** A retrospective cohort study was performed looking at patients who underwent delayed or immediate autologous breast reconstruction with a DIEP flap over a 6-year period (2015-2021) at our institution. Complication rates for abdominal infection, seroma, hematoma, wound dehiscence, delayed wound healing, umbilical necrosis, subjective abdominal weakness, abdominal bulge, and hernia were calculated. Additionally, a prospective cohort study was conducted using a portable ultrasound device to detect postoperative changes in the abdominal donor site, including fluid collections and postoperative edema. **Results:** One hundred seventeen patients underwent autologous breast reconstruction with a DIEP-free flap. Forty-one percent of patients experienced 1 or more donor site complications. Complication rates were 16.2%, 12.8%, 1.7%, 15.4%, 8.5%, 4.3%, 0%, 10.3%, and 2.6%, respectively, for the list above. There was a higher proportion of complications in patients who smoked within the past 3 months and those who had a body mass index (BMI) between 35 and 39.9, although this was not statistically significant. Bilateral reconstructions had higher rates of umbilical necrosis (24.5% vs 7.8%) and wound dehiscence (9.4% vs 0%) compared to unilateral. Twenty-one patients were included in the prospective analysis. No significant changes in abdominal wall edema were found. Twenty-four percent of the patients had detectible collections on ultrasound, and these were associated with wound dehiscence and the need for debridement. **Discussion:** Our institutional abdominal donor site complication rates in DIEP reconstruction patients are higher than those published in the literature. Similar complication rates were identified regardless of smoking status, BMI, and unilateral/bilateral surgery. Quality improvement initiatives could be considered and implemented to reduce future complications.

## Résumé

**Historique:** Le lambeau libre de l’artère épigastrique inférieure profonde (AÉIP) est le plus utilisé pour la reconstruction mammaire autologue. Malgré la réduction relative des complications au foyer du donneur par rapport aux options sans épargne musculaire, cette opération entraîne tout de même une morbidité élevée. La présente étude visait à comparer les résultats et les complications à l’établissement des chercheurs et à exposer les initiatives potentielles d’amélioration de la qualité. **Méthodologie:** Les chercheurs ont réalisé une étude de cohorte rétrospective auprès de patientes qui ont subi une reconstruction mammaire autologue immédiate ou tardive au moyen d’un lambeau de l’AÉIP sur une période de six ans (2015 à 2021) à leur établissement. Ils ont calculé les taux de complication par infection abdominale, sérome, hématome, déhiscence de plaie, retard de la cicatrisation, nécrose ombilicale, faiblesse abdominale, renflement abdominal ou hernie. De plus, ils ont effectué une étude de cohorte prospective au moyen d’un système d’échographie portatif pour déceler les changements postopératoires au foyer abdominal du donneur, y compris la collecte de liquide et l’œdème postopératoire. **Résultats:** Au total, 117 patientes ont subi une reconstruction mammaire autologue par lambeau libre de l’AÉIP. Ainsi, 41% des patientes ont subi au moins une complication au foyer du donneur. Par rapport à la liste précédente, les taux de complication correspondaient à 16.2%, 12.8%, 1.7%, 15.4%, 8.5%, 4.3%, 0%, 10.3% et 2.6%, respectivement. La proportion de complications était plus élevée chez les patientes qui avaient fumé dans les trois mois précédents et chez ceux dont l’indice de masse corporelle se situait entre 35 et 39,9, même si cette observation n’était pas statistiquement significative. Les reconstructions bilatérales étaient associées à un plus fort taux de nécrose ombilicale (24.5% par rapport à 7.8%) et de déhiscence des plaies (9.4% par rapport à 0%) que les reconstructions unilatérales. Dans l’ensemble, 21 patientes ont participé à l’analyse prospective. Les chercheurs n’ont observé aucun changement important à l’œdème de la paroi abdominale. Par ailleurs, 24% des patientes avaient des épanchements décelables à l’échographie, associés à une déhiscence des plaies et à la nécessité d’un débridement. **Discussion:** Le taux de complications au foyer abdominal du donneur chez les patientes qui ont subi une reconstruction par AÉIP est plus élevé dans l’établissement des chercheurs que celui indiqué dans les publications scientifiques. Les chercheurs ont déterminé des taux de complication semblables quels que soient le tabagisme, l’indice de masse corporelle et la chirurgie unilatérale ou bilatérale. On pourrait envisager et mettre en œuvre des initiatives d’amélioration de la qualité pour réduire les futures complications.

## Introduction

The deep inferior epigastric perforator (DIEP) flap is the most commonly used free flap in autologous breast reconstruction.^1,2^ The use of DIEP flaps for breast reconstruction was first described in 1994^3^ and has since become the gold standard due to pleasing aesthetic outcomes and reduced morbidities in comparison to other flaps, such as the transverse rectus abdominis muscle (TRAM) flap. Although DIEP flap harvest is more time-consuming and technically challenging, preserving the rectus abdominus muscle significantly reduces donor site complications such as abdominal hernias and fat necrosis relative to TRAM flaps.^4,5^ Patient satisfaction has also been reported to be higher after reconstruction with DIEP flaps compared to TRAM flaps.^6^

Although there are relatively fewer donor site complications in DIEP patients in comparison to nonmuscle-sparing options, donor site complication rates remain high. Several groups have studied donor site complications in DIEP patients and saw high incidences of dehiscence and delayed wound healing up to 39%, seromas up to 48%, and abdominal bulging up to 7%.^7–9^ Some patient-specific risk factors that have been associated with increased rates of complications include obesity, diabetes mellitus, tobacco use, or chemotherapy.^10–13^ Abdominal complications following DIEP flap harvest can result in significant patient morbidity and distress, as well as affecting patient satisfaction, body image, and quality of life.^8,14^

While there are many reviews on abdominal complications reported in the literature, specific center-dependant or demographic factors may influence regional complication rates. The purpose of this study is to examine the rates of DIEP donor site abdominal complications at our institution both retrospectively and prospectively, as a means of quality assurance. Ultimately, we hope this information can be used to provide patients with improved informed consent and to serve as a baseline for future quality improvement initiatives.

## Materials and Methods

The retrospective portion of this study was approved by the Research Ethics Board (REB #118731). A list of patients who underwent autologous reconstruction with a DIEP flap from January 1, 2015 to February 24, 2021 was identified by searching the R066 OHIP billing code submitted by the 3 current reconstructive breast microsurgeons at our institution. Those who underwent immediate or delayed autologous breast reconstruction with unilateral or bilateral free DIEP flap reconstruction were included. Patients were excluded from our study if they underwent autologous breast reconstruction with free tissue transfer other than a DIEP flap (eg, TRAM flaps were excluded).

Charts of the identified patients were reviewed for basic demographic information, past medical history (including type 2 diabetes, smoking status in the 12 months prior to consent for surgery), previous abdominal surgeries, operative details, and duration of follow-up. Abdominal donor site complications were recorded including infection (fevers, chills, peri-incisional cellulitis, purulent discharge), seroma, hematoma, wound dehiscence, delayed wound healing, umbilical necrosis, subjective abdominal weakness, bulge and hernia.

Descriptive statistics were completed using Microsoft Excel. Fisher's exact test and ANOVA were used to compare outcomes based on smoking status, DIEP type (unilateral vs bilateral), and body mass index (BMI) group. These comparison groups were determined a priori. Posthoc testing was not required as none of these values were statistically significant.

The prospective study portion was approved by the Research Board (REB 119945). Similarly, patients undergoing unilateral or bilateral DIEP flap between February 2022 to January 2023 were included. Since one of the outcomes of interest was abdominal edema, individuals with significant medical history that could confound results, such as congestive heart failure and renal disease, were excluded. A basic chart review to obtain basic demographic information and pertinent postoperative complications was completed.

For this portion of the study, we utilized a portable point-of-care ultrasound (POCUS) machine, Butterfly IQ+^®^ (Butterfly Network, Inc). This was used to measure the abdominal flap thickness immediately after surgery and at their first postoperative follow-up (2-6 weeks postoperative), to detect any significant changes. All measurements were made 3 cm above the newly positioned umbilicus from skin to rectus fascia, to maintain consistency. This point was chosen for the detection of edema as it was approximately the mid-point of the raised abdominal flap, between the incision and the costal margin. POCUS was also used to detect any fluid collections not clinically assessable.

## Results

### Retrospective Study

One hundred seventeen patients were identified to have undergone immediate or delayed, unilateral or bilateral breast reconstruction with free DIEP flaps. The mean BMI was 30.5 ± 0.5. Six patients reported having smoked within the 12 months prior to surgery. Follow-up period was 412.6 ± 35.7 days. Unilateral DIEP reconstructions took an average of 450 ± 8 min, while bilateral reconstructions took 637 ± 12 min.

Forty-one percent (*n* = 48) of patients experienced 1 or more complications, such as infection, seroma, hematoma, wound dehiscence, subjective abdominal weakness, abdominal bulge, abdominal hernia, delayed wound healing and umbilical necrosis. These results are summarized in Table 1.

**Table 1.** Total Percentage of Patients With Abdominal Donor Site Complication (*n* = 117) Compared to Published Meta-Analyses.

	Our institution (*n* = 117)	Man et al^17^	He et al^6^	Jeong et al^18^	Salgarello et al^19^	Wang et al^20^
Infection	16.2				4.9 (*n* = 571)	2.7 (*n* = 74)
Seroma	12.8				3.7 (*n* = 1940)	7.2 (*n* = 125)
Hematoma	1.7					8.1 (*n* = 307)
Wound dehiscence	15.4				7.2 (*n* = 510)	
Delayed wound healing	8.5		37.3 (*n* = 284)			
Umbilical necrosis	4.3				4.8 (*n* = 1168)	
Abdominal weakness	0					
Abdominal bulge	10.3	3.1 (*n* = 930)	7.4 (*n* = 1000)	3.9 (*n* = 1211)		5.9 (*n* = 508)
Hernia	2.6	0.8 (*n* = 1529)				2.0 (*n* = 391)

**Figure 1.**
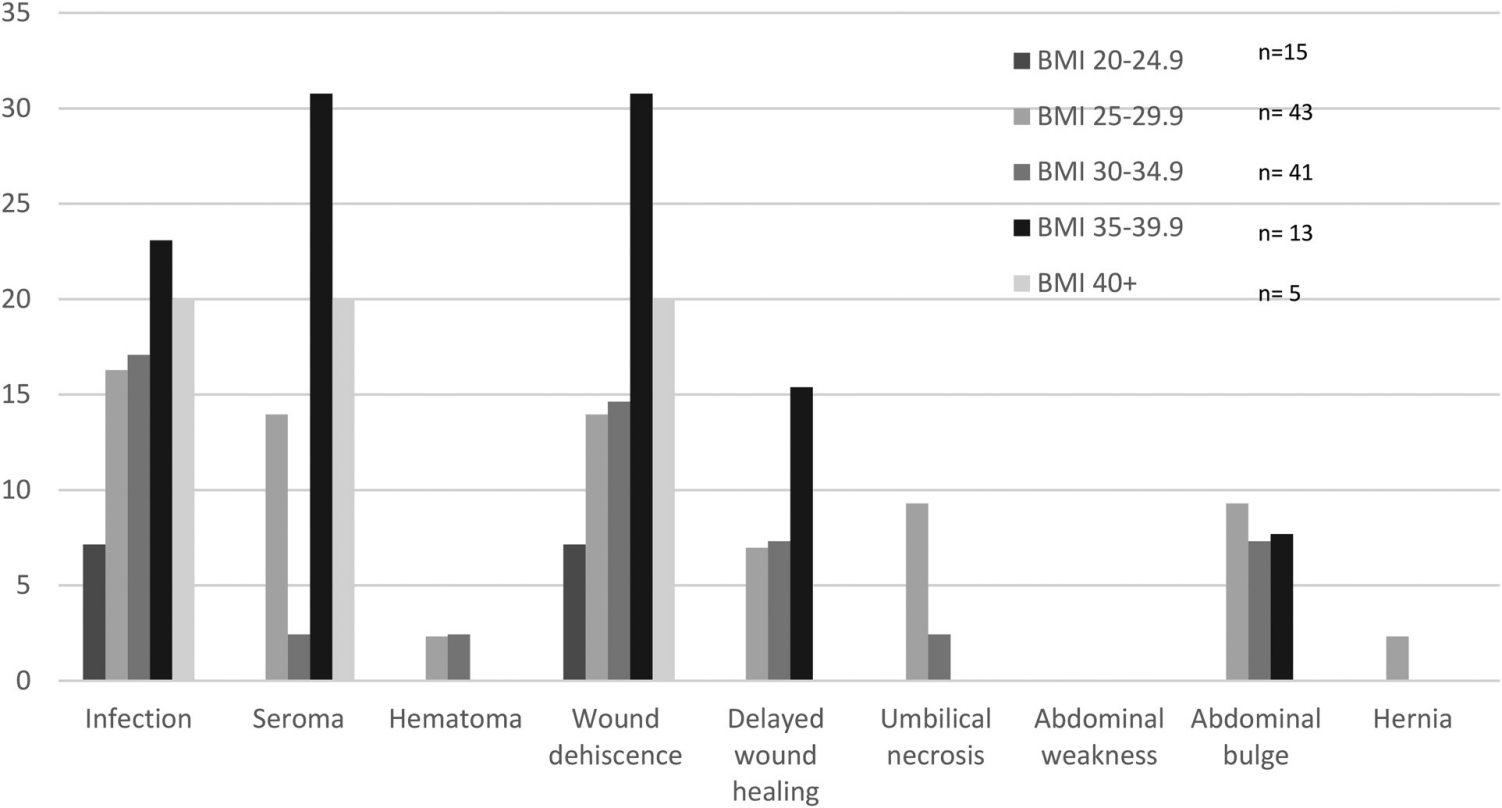
Percentage of patients with abdominal donor site complications stratified by BMI.

The majority (94.5%) of patients were nonsmokers. There was a greater proportion of infection, seroma, wound dehiscence, abdominal bulge, and umbilical necrosis in the smoking population, although these did not reach statistical significance (Table 2). When patients were stratified by BMI, those between 20 and 24.9 had the lowest complication rates overall, while those between 35 and 39.9 had the highest overall complication rates, although these were not statistically significant (Figure 1).

BMI, body mass index.

**Table 2.** Abdominal Donor Site Complication Rates Stratified by Smoking Status in the Previous 12 Months Prior to Surgery.

	Smokers (*n* = 6)		Nonsmokers (*n* = 111)		*P*-value
	Number of patients	Percentage (%)	Number of patients	Percentage (%)	
Infection	2	33.3	17	15.3	.25
Seroma	2	33.3	13	11.7	.17
Hematoma	0	0	2	1.8	1
Wound dehiscence	1	16.7	17	15.3	1
Delayed wound healing	0	0	10	9.0	1
Umbilical necrosis	1	16.7	4	3.6	.49
Abdominal weakness	0	0	0	0	1
Abdominal bulge	1	16.7	11	9.9	1
Hernia	0	0	3	2.7	.24

**Figure 2.**
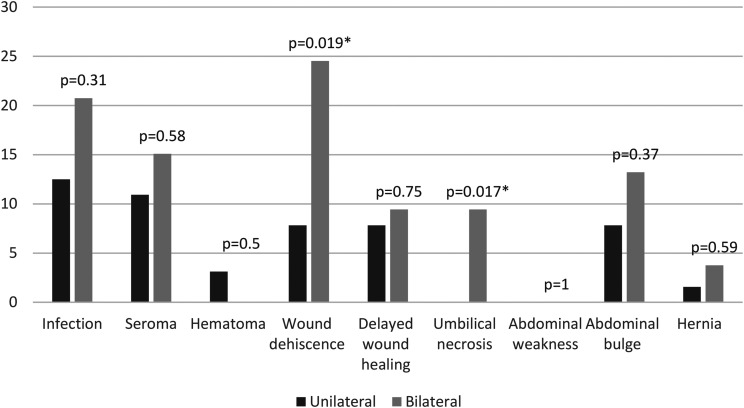
Percentage of patients with abdominal donor site complications in unilateral versus bilateral DIEP patients.

Patients who underwent bilateral DIEP reconstruction had statistically significant higher rates of wound dehiscence (*P* = .019) and umbilical necrosis (*P* = 0.017) than those who had unilateral reconstruction (Figure 2).

DIEP, deep inferior epigastric perforator artery.

* signifies statistical significance *P* < .05.

### Prospective Study

Twenty-one patients consented to participation in the study, and 17 patients were seen postoperatively at their clinic appointment in 2-3 weeks, except 1 patient whose follow-up was at 6 weeks. General complication rates were followed for 6 weeks. The mean age of the participants was 48.8, and the mean BMI was 30.4. Bilateral DIEP was more common, at 61% of all surgeries. Only one of the patients had a smoking history in the past 12 months.

In terms of outcomes, 1 patient had abdominal donor site infection, and 3 patients had wound dehiscence. Some of these patients required wound care by community nursing for up to 6 weeks. One patient developed an abdominal hematoma that required debridement. Four out of the 17 patients (23.5%) who had a POCUS assessment of their abdomen had a detectible fluid collection. Among the 4 patients who had detectible collection, 1 had the above-mentioned hematoma, 1 had wound dehiscence, and 1 had not been wearing a binder. The results are summarized in Table 3.

**Table 3.** Abdominal Donor Site Changes and Complication Rates in the First 6 Weeks of Surgery Followed Prospectively.

	Total participants (*n* = 21)	
	Number of patients	Percentage (%)
Infection	1	4.8
Hematoma	1	4.8
Seroma (assessed clinically)	2	9.5
Subjective swelling	2	9.5
Wound dehiscence	3	14.3
Need for wound care at 6 weeks	2	9.5
Umbilical necrosis	0	0
Collection (assessed on POCUS at 2 weeks)	4	23.5 (*n* = 17)

POCUS, point-of-care ultrasound.

**Figure 3.**
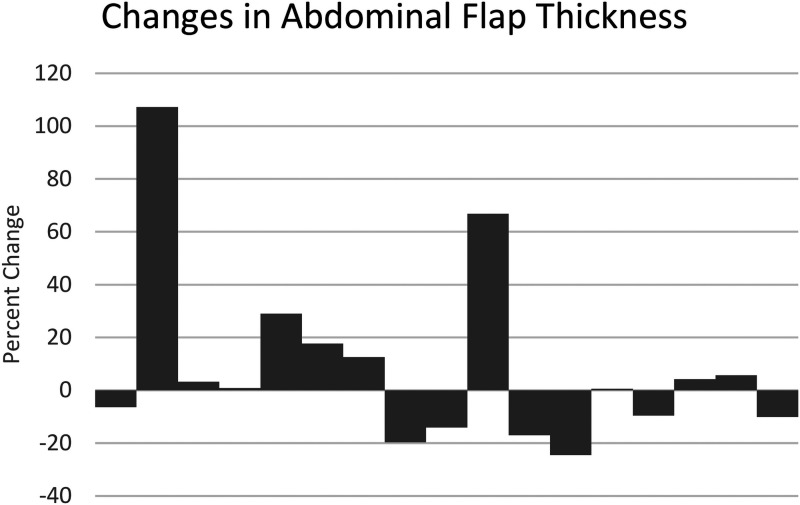
Percent change in abdominal flap thickness measured by POCUS at postoperative follow-up, post-DIEP-flap-based breast reconstruction.

*Note:* The average change was +8.6%, but when the outliers were removed, the change was −1.8%. POCUS, point-of-care ultrasound; DIEP, deep inferior epigastric perforator artery.

The abdominal tissue thickness was also measured using the POCUS. Overall, there was minimal change (+8.6%) change in the abdominal tissue thickness at the 2 to 6-week follow-up mark. Because of the methodology of measuring thickness from skin to rectus fascia, those who developed fluid collection between the abdominal skin and the fascial layer were noticeable outliers. With the outliers removed, the change was further reduced at −1.8%. The bar graph visualizing individual change is shown in Figure 3.

## Discussion

The number of autologous breast reconstructions performed at our institution has steadily increased year over year. DIEP-flap-based breast reconstruction remains popular for its advantage of using autologous tissue, and having favorable aesthetic outcomes in both the donor and recipient sites. This procedure, however, is not without risks. The surgeries are long, ranging from 7.5 to 10.6 h as seen in our study, and can result in complications at both the donor and recipient sites.

This study investigates the abdominal donor site complication rates in the DIEP flap-based breast reconstruction population within our institution. This study is unique as we implemented both retrospective, and prospective components to review and follow patient outcomes after DIEP flap-based breast reconstruction. In our review, we identified that 41% of our patients experienced one or more of the investigated complications. In our prospective analysis, again, a high incidence of fluid collection and wound healing issues were identified. These are despite the use of routine perioperative antibiotics (cefazolin 2 grams intravenous within 30 min of skin incision, or clindamycin 600 mg intravenous within 30 min of skin incision) and intraoperative redosing every 4 h. In addition, routine practice includes two 19-french Blake drains that exit the mons, meticulous hemostasis of the wound, irrigation, and standardized layered abdominal closure with 4-0 vicryl for muscle repair, 0 PDS to close fascia, 2-0 vicryl to repair Scarpa's fascia, 3-0 monocryl to close the dermis, followed by a 4-0 monocryl in a running subcuticular fashion.

In our review of 117 patients, complication rates compared between smokers and nonsmokers were not statistically different, which is inconsistent with the literature.^13,15,16^ This could be the result of the cohort being small, and possible discrepancy in patients admitting to smoking within 3 months of surgery. Increased complication risks associated with smoking are routinely counselled during consultation, and smoking cessation is encouraged preoperatively.

There are confounding factors that may have contributed to our high complication rates, including patient comorbidities that impair wound healing such as diabetes, relative malnutrition postcancer treatment, and neoadjuvant chemotherapy. In addition, this patient population is generally heterogeneous. This is a strength of our study as our wide inclusion criteria of patients who underwent DIEP free flap reconstruction is representative of the true patient population, and provides us with a realistic risk profile to discuss when counselling patients preoperatively.

We compared our results with the current literature, particularly meta-analyses that examined abdominal complication rates after DIEP-based reconstruction and similar surgeries such as TRAM and elective abdominoplasty.^6,17–20^ While the selected meta-analyses showed heterogeneous groups of patients with potentially different abdominal closure methods such as the use of abdominal mesh, it is apparent that our institutional complication rates for abdominal donor sites are higher. For instance, a meta-analysis of donor site infection rates ranged from 2.7 to 4.9% in our search,^19,20^ whereas our institution's infection rate was 16.2%. Abdominal bulge also happened at higher rates at 10.3%, relative to 3.1∼7.4% seen in the literature.^6,17^ This highlights the importance of performing interim analyses on surgical outcomes, as it provides room for improvement and prompt intervention as necessary.

In the prospective examination of our patients, we decided to adopt POCUS to better characterize aspects that are not always clinically evident, in addition to the more clinically apparent complications such as wound dehiscence. These included the presence of seroma or hematoma under the abdomen and changes in the abdominal flap thickness representing postoperative edema. Since many patients are concerned about swelling or edema for the first few weeks, we wanted to see if an ultrasound would be a good way to measure the flap thickness change. We measured from the skin to the rectus fascia where the rectus muscle joins into linea alba. While some images were of high quality, others were confounded by rectus diastasis, which made it difficult to find the correct anatomy. This study demonstrated that POCUS can be a valuable resource for clinicians to assess postoperative outcomes in DIEP breast reconstruction patients, and as a tool for future research to see efficacy of specific interventions.

The results of our study were discussed with the 3 reconstructive breast surgeons at our institution and also in our division-based research meeting. Potential changes to minimize negative outcomes that could be easily implemented in our current protocol were widely discussed. These include more stringent patient selection for surgery with strict BMI cut-offs, smaller abdominal tissue for harvest to result in a closure with reduced tension, adjunctive tools such as incisional negative pressure wound therapy (NPWT) and progressive tension sutures, as well as efficient use of operative time.

Limitations to our study include the small sample size and the retrospective nature of the study. In total, we examined the outcomes of 134 patients, from a single institution. Although this is valuable for quality improvement among our patient group, it may not be necessarily generalizable to the rest of the population undergoing DIEP-flap-based breast reconstruction. With the retrospective study design, we found it challenging to decipher our outcome measures clearly (for instance, wound dehiscence vs infection) and required interpretation of the clinical note in its entire context during the chart review. Additionally, the outcome measure “subjective abdominal weakness” was difficult to assess retrospectively as it is unlikely that every patient was asked specifically at each visit. Another limitation is that we lack patient-reported outcomes, such as the BREAST-Q Questionnaire.^21,22^ Failure to administer the BREAST-Q to our patients was identified as a significant problem, and since July 2021 we started to obtain these from all autologous and alloplastic breast reconstructive patients of our institution.

There are other factors that were not included in our analysis but can be considered in future analysis. For instance, we did not examine the number of the perforators used, and which row they were taken. Current literature suggests that the location and the number of perforators can affect donor site morbidity.^8^ We also did not extract information on whether an attempt of superficial inferior epigastric vein (SIEV) dissection was made. Additional time spent on SIEV dissection can negatively impact operating time, and increase donor complication rates.^23,24^

There are several interventions that have been identified in the literature that may reduce the risks of postoperative donor site complications. Incorporating abdominal mesh during fascial closure of the donor site has been shown to reduce the incidence of hernias and abdominal bulges.^25,26^ Use of biologic or synthetic mesh is not routinely performed at our institution but can be considered and used in a suitable patient, especially if fascial closure is excessively tight. Umbilical ablation at the time of harvest has also been explored as a method for reducing abdominal complications.^27^ The use of progressive tension sutures to close the dead space that is created with the undermining of the abdominal flap is used routinely in aesthetic surgery and may be beneficial in the reconstructive setting as well.^28–30^ This would especially be helpful to reduce the high rates of fluid collection we’ve seen in the prospective portion of the study. By the first postoperative follow-up, which occurs 2-3 weeks after the operation, all drains are removed. The drains are removed when output is less than 30 cc for 2 consecutive days, and are typically removed between postoperative days 7 to 10. Patients are also provided with an abdominal binder to wear for 6 weeks postoperatively. Despite these measures, we frequently saw collections due to the dead space that is created, and they were associated with other complications such as wound dehiscence. Closed incision NPWT has also been shown to reduce complications such as wound dehiscence, infection and delayed wound healing.^31–36^ At this time, closed incision NPWT is not routinely practiced in our institution, however, we are planning a randomized controlled trial to compare the donor site outcomes in those who receive incisional NPWT postoperatively versus those receiving standard dressings.

Moving forward, there remains room for further research in optimizing the outcomes and recovery of DIEP surgery patients. Our results again emphasize the need for interventions to reduce common donor site complications as much as possible.

## Presentations

This study was presented at the Division of Plastic and Reconstructive Surgery Research Day, and not published elsewhere.
